# FGFR-gerichtete Therapie von Kopf-Hals-Karzinomen

**DOI:** 10.1007/s00106-020-00893-2

**Published:** 2020-06-09

**Authors:** Dimo Dietrich

**Affiliations:** Klinik und Poliklinik für Hals-Nasen-Ohrenheilkunde/Chirurgie, Universitätsklinikum Bonn, Venusberg-Campus 1, 53127 Bonn, Deutschland

**Keywords:** Fibroblasten-Wachstumsfaktor-Rezeptor, Tyrosinkinaseinhibitor, Plattenepithelkarzinom des Kopf-Hals-Bereichs, Prädiktiver Biomarker, Mutation, Fibroblast growth factor receptor, Tyrosine kinase inhibitor, Head and neck squamous cell carcinoma, Predictive biomarker, Mutation

## Abstract

**Hintergrund:**

Genomische Veränderungen (Mutationen, Genfusionen, Amplifikationen) und eine Dysregulierung des Fibroblasten-Wachstumsfaktor-Rezeptor-Signalwegs („fibroblast growth factor (FGF) receptor“, FGFR) sind häufig in Plattenepithelkarzinomen des Kopf-Hals-Bereichs (HNSCC) zu finden. Eine zielgerichtete Therapie mit gegen FGF-Rezeptoren gerichteten Tyrosinkinaseinhibitoren (TKI) oder monoklonalen Antikörpern stellt daher einen vielversprechenden Ansatz für die Behandlung des HNSCC dar.

**Fragestellung:**

Dieser Übersichtsartikel beschreibt den aktuellen Wissensstand hinsichtlich FGFR-gerichteter Therapien bei Kopf-Hals-Tumoren (insbesondere HNSCC) und diskutiert in diesem Kontext genomische Veränderungen des FGFR-Signalwegs als potenzielle begleitende prädiktive Biomarker.

**Material und Methode:**

Der vorliegende Artikel basiert auf einer Recherche der Datenbanken PubMed, ClinicalTrials.gov sowie von Tagungsbänden.

**Ergebnisse:**

Erste Ergebnisse belegen die Wirksamkeit von Tyrosinkinase-Inhibitoren sowohl bei HNSCC als auch bei Adenokarzinomen des Kopf-Hals-Bereichs, insbesondere bei Schilddrüsenkarzinomen und adenoid-zystischen Speicheldrüsenkarzinomen.

**Schlussfolgerungen:**

Frühe klinische und präklinische Daten verdeutlichen das Potenzial einer biomarkergesteuerten Behandlung von Patienten mit Kopf-Hals-Tumoren mit gegen FGFR-gerichtete TKI.

## Abstract (en)

**Background:**

Genomic aberrations (mutations, gene fusions, amplifications) and dysregulation of the fibroblast growth factor (FGF) receptor (FGFR) signaling pathway are frequently found in squamous cell carcinomas of the head and neck (HNSCCs). Targeted therapy with tyrosine kinase inhibitors (TKIs) or monoclonal antibodies directed against FGF receptors therefore represents a promising approach for the treatment of HNSCC.

**Objective:**

This review article describes the current status of FGFR-directed therapies for head and neck tumors (especially HNSCC) and, in this context, discusses genomic alterations of the FGFR pathway as potential companion predictive biomarkers.

**Methods:**

This article is based on searches of PubMed, ClinicalTrials.gov, and conference proceedings.

**Results:**

First results prove the efficacy of TKIs both in HNSCC and in adenocarcinomas of the head and neck, especially in thyroid and adenocystic salivary gland carcinomas.

**Conclusion:**

Early clinical and preclinical data point to the promise of biomarker-directed treatment of patients with head and neck tumors using FGFR-targeted TKIs.

Biomarkergesteuerte zielgerichtete Therapien, insbesondere die Inhibierung von Tyrosinkinasen, stellen einen wirksamen Therapieansatz für fortgeschrittene maligne Erkrankungen dar. Therapeutische monoklonale Antikörper (mAb) und TKI haben bereits den Weg in die leitliniengemäße Behandlung verschiedener maligner Erkrankungen gefunden. So ist in der EU bereits seit mehr als einem Jahrzehnt der gegen den epidermalen Wachstumsfaktorrezeptor („epidermal growth factor receptor“, EGFR) gerichtete Antikörper Cetuximab für die Behandlung fortgeschrittener HNSCC zugelassen. Kürzlich erhielt der FGFR-Inhibitor Erdafitinib in den USA nach hervorragenden klinischen Ergebnissen die beschleunigte Zulassung für die Behandlung fortgeschrittener oder metastasierter Urothelkarzinome mit genetischen Veränderungen von *FGFR2* oder *FGFR3*. Eine Dysregulierung der FGFR-Signalkaskade ist häufig bei HNSCC zu finden und könnte daher auch hier ein Ziel einer zielgerichteten Therapie darstellen.

## Die Erfolgsgeschichte der Tyrosinkinaseinhibitoren

Seit den 1980er-Jahren wird die Rolle von Rezeptortyrosinkinasen (RTK) bei der Entwicklung, Progression und Metastasierung von malignen Erkrankungen intensiv untersucht. RTK sind zellmembrangebundene Rezeptoren für eine Reihe von Wachstumsfaktoren, Zytokinen und Hormonen mit einer intrazellulären Kinaseaktivität, welche die Phosphorylierung von Tyrosinresten und damit eine Signaltransduktion ermöglicht. Über RTK werden vielfältige zelluläre Funktionen, wie beispielsweise Proliferation, Embryonalentwicklung, Zelldifferenzierung, Angiogenese und Wundheilung, reguliert. Entsprechend diesen Funktionen kann eine Dysregulierung von RTK zu Tumorentstehung, verstärktem Wachstum und Metastasierung führen. Aus dem Verständnis über die Bedeutung von RTK in der Onkogenese resultierte das therapeutische Konzept, RTK mittels mAb oder kleiner Moleküle (sogenannter TKI) selektiv und spezifisch zu inhibieren [1]. Dies führte zu einer Reihe von Medikamentenzulassungen, wie beispielsweise dem mAb Trastuzumab (Herceptin®, F. Hoffmann-La Roche AG, Basel, Schweiz) Ende der 1990er-Jahre für die Behandlung von Patientinnen und Patienten mit metastasiertem Mammakarzinom, deren Tumoren eine Überexpression der RTK HER-2/neu („human EGFR 2“, offiziell bekannt als ERBB2 [„erb-b2 receptor tyrosine kinase 2“]) aus der Familie der EGFR aufweisen. In der EU folgte im Jahr 2006 die Zulassung des gegen EGFR (ERBB1) gerichteten mAb Cetuximab (Erbitux®, Merck KGaA, Darmstadt, Deutschland) für die Behandlung fortgeschrittener HNSCC in Kombination mit einer Bestrahlung. Seit 2008 ist das Präparat für die Erstlinientherapie von rezidivierenden und/oder metastasierenden HNSCC in Kombination mit einer platinbasierten Chemotherapie zugelassen. Seit Anfang der 2000er-Jahre wurden weitere TKI für die Behandlung verschiedener maligner Erkrankungen zugelassen, die Zulassung eines TKI für die Monotherapie des HNSCC steht jedoch bislang aus. Klinische Studien und Erfahrungen aus dem klinischen Alltag belegen zum Teil beeindruckende Behandlungserfolge durch TKI. Dieses wertvolle therapeutische Potenzial sollte neben der aktuellen Euphorie bezüglich der Wirksamkeit von Immuncheckpointinhibitoren nicht in den Hintergrund treten. Die Wirksamkeit von TKI ist besonders dann hoch, wenn anhand genetischer Veränderungen des Zielmoleküls ein klinisches Ansprechen akkurat vorausgesagt werden kann. Die kritische Selektion eines passenden Patientenkollektivs ist damit für den Behandlungserfolg entscheidend. Beispielsweise ist bei der chronisch myeloischen Leukämie (CML) die „Abelson murine leukemia“ (ABL)-Tyrosinkinase ursächlich dauerhaft aktiviert und dysreguliert. Der Einsatz des ABL-Tyrosinkinase-Inhibitors Imatinib (Glivec®, Novartis AG, Basel, Schweiz) führte zu sehr guten Behandlungserfolgen und daher 2001 zur ersten Zulassung eines TKI in Europa. Imatinib ist heutzutage Standard bei der Behandlung der CML. Der TKI Alectinib (Alecensa®, F. Hoffmann-La Roche AG) erreichte eine Ansprechrate von 82,9 % bei Patienten mit zuvor unbehandelten fortgeschrittenen nichtkleinzelligen Lungenkarzinomen, deren Tumoren eine Mutation in der RTK „anaplastic lymphoma kinase“ (*ALK*) trugen [2]. Ein weiteres Beispiel ist der TKI Larotrectinib (Vitrakvi®, Bayer AG, Leverkusen, Deutschland), der bei Patienten, deren Tumoren eine Genfusion unter Beteiligung einer der drei NTRK („neurotrophic receptor tyrosine kinase“) trägt, Ansprechraten von 75–80 % zeigte [3]. Mit der US-Zulassung von Larotrectinib für alle NTRK-Genfusion-tragenden Tumorerkrankungen wird der Paradigmenwechsel bei der biomarkergeleiteten Wirkstoffentwicklung weitergeführt: Nach der Zulassung des Anti-PD-1-Immuncheckpointinhibitors Pembrolizumab (Keytruda®, Merck Sharp & Dohme, Kenilworth, NJ, USA) für alle mikrosatelliteninstabilen und Mismatch-Reparatur-defizienten Tumoren stellt Larotrectinib die nächste histologieunabhängige Zulassung auf Basis einer genetischen Veränderung dar. Somit steht Larotrectinib grundsätzlich für eine Behandlung von Kopf-Hals-Tumorpatienten in den USA zur Verfügung, sofern diese Tumoren eine NTRK-Genfusion tragen. Während diese Mutation bei Speicheldrüsentumoren relativ häufig ist und die Tumoren ein dementsprechend gutes Therapieansprechen auf Larotrectinib zeigen [3], ist eine NTRK-Genfusion bei HNSCC – wenn überhaupt – nur äußerst selten zu finden. So konnte in der HNSCC-Kohorte des *The Cancer Genome Atlas* nur in einem Tumor (0,3 % aller untersuchter Fälle) eine NTRK-Genfusion gefunden werden [4]. Wesentlich häufiger sind bei HNSCC die Mitglieder der FGFR-RTK-Gene *FGFR1, FGFR2* und *FGFR3* von Mutationen betroffen [5, 6] und stellen daher ein vielversprechendes Ziel für eine spezifische Inhibierung mit mAb oder TKI bei HNSCC dar. Für den FGFR-Inhibitor Erdafitinib (Balversa®, Janssen Pharmaceutica N.V., Beerse, Belgien) konnte in einer Phase-II-Studie bei Patienten mit lokal fortgeschrittenen oder metastasierten Urothelkarzinomen mit zuvor spezifizierten genetischen Veränderungen der FGFR-Gene eine Ansprechrate von 40 % gezeigt werden [7]. Bei Patienten, die zuvor bereits eine Immuntherapie erhalten hatten, lag die Ansprechrate mit 59 % sogar besonders hoch [7]. Derzeit werden weitere Studien mit FGFR-modulierenden Wirkstoffen durchgeführt, und Ergebnisse sind in Kürze zu erwarten. Ziel dieses Artikels ist es, einen Überblick über die aktuelle Anwendung von FGFR-gerichteten mAb und TKI in der Behandlung von HNSCC und weiteren malignen Erkrankungen des Kopf-Hals-Bereichs zu liefern.

## Der FGFR-Signalweg

Die FGFR-Genfamilie umfasst unter anderem die vier Mitglieder *FGFR1*–*4*, die eine hohe Sequenzhomologie von 55–72 % aufweisen [8]. Alle vier FGFR-Gene zeigen einen ähnlichen strukturellen Aufbau, mit einer extrazellulären Ligandenbindestelle, einer einfachen Transmembran-Domäne und einer intrazellulären Kinase-Domäne [9]. Einem weiteren Mitglied der Genfamilie, FGFR5 (auch als *FGFRL1* bezeichnet), fehlt die intrazelluläre Kinase-Domäne [10]. Bei FGFR6 (auch als *FGFR3P1* bezeichnet) handelt sich um ein *FGFR3*-Pseudogen, dem bisher keine Funktion zugeschrieben werden konnte. Alternatives Spleißen der FGFR1–3-Transkripte führt darüber hinaus zur Bildung multipler Isoformen, mit zum Teil stark unterschiedlicher Ligandenbindespezifität [11, 12].

Die natürlichen Liganden der FGFR sind die FGF. Insgesamt umfasst die FGF-Familie 22 bekannte Familienmitglieder (FGF1–14 und FGF16–23). FGF lassen sich weiter in sechs Unterfamilien unterteilen. Die FGF1- (FGF1 und FGF2), FGF4- (FGF4–FGF6), FGF7- (FGF3, FGF7, FGF10 und FGF22), FGF8- (FGF8, FGF17 und FGF18) und die FGF9-Unterfamilie (FGF9, FGF16 und FGF20) wirken parakrin, die FGF19-Unterfamilie (FGF19, FGF21 und FGF23) wirkt endokrin [13]. FGF11–FGF14 weisen zwar ein hohes Maß an Homologie zu den anderen FGF auf, sind aber nicht in der Lage, FGFR zu aktivieren [14].

Der FGFR-Signalweg ist in einigen exzellenten Übersichtsarbeiten im Detail anschaulich erläutert und wird im Folgenden nur kurz beschrieben [12, 15–17]. Nach Bindung der FGF folgt eine Dimerisierung der FGFR, gefolgt von einer Transautophosphorylierung und anschließender Aktivierung nachgeschalteter Signalwege, beispielsweise des „mitogen-activated protein kinase“ (MAPK)- und des Phosphoinositid-3-Kinasen (PI3K)/Proteinkinasen B (AKT)-Signalwegs [12]. Diese Signalwege sind an der Embryogenese, der Zelldifferenzierung, der Migration, der Angiogenese, dem Zellwachstum und dem programmierten Zelltod beteiligt. Aufgrund der Aktivierung dieser bei der Tumorentstehung und Progression relevanten Signalwege stellen die FGFR typische Onkogene und ein ideales Ziel für gerichtete medikamentöse Therapien dar.

## Dysregulierung des FGFR-Signalwegs bei malignen Erkrankungen

Eine Dysregulierung von FGFR-gesteuerten Signalwegen ist in vielen Tumoren, insbesondere bei HNSCC, bei Schilddrüsenkarzinomen und adenoid-zystischen Speicheldrüsentumoren, zu finden und spielt eine treibende Rolle, beispielsweise bei der Angiogenese [16, 18, 19]. Eine Reihe von Veränderungen in FGFR-Signalwegen sind bei malignen Erkrankungen relevant: Eine Genamplifikation oder eine posttranskriptionelle Regulation kann zu einer Überexpression der Rezeptoren führen; Mutationen können zu einer konstitutiven Rezeptoraktivierung oder einer geringeren Liganden-Abhängigkeit führen; nach Translokationen können Fusionsproteine mit einer konstitutiven Aktivität gebildet werden; eine Überexpression der FGF kann eine parakrine oder autokrine Aktivierung von FGFR-Signalwegen induzieren, und alternatives Spleißen kann die Liganden-Spezifität verändern [20].

### Überexpression

Eine Überexpression kann zu einer ligandenunabhängigen Dimerisierung der FGFR und damit zu einer Aktivierung der FGFR-Signalkaskade führen [21]. Eine Genamplifikation ist ein wesentlicher Faktor, der zu einer FGFR-Überexpression führt. FGFR-Genamplifikationen sind in manchen Tumorentitäten sehr häufig zu finden [12, 16]. Eine *FGFR1*-Amplifikation ist beispielsweise je nach Studie für 8–22 % der Plattenepithelkarzinome der Lunge beschrieben [22, 23]. In 279 HNSCC-Tumoren des *The Cancer Genome Atlas* konnten FGFR-Amplifikationen in 27 der Tumoren (9,7 %) nachgewiesen werden. Dabei traten FGFR-Amplifikationen ausschließlich in den 243 untersuchten human Papillomvirus (HPV)-negativen Tumoren auf und betrafen vorwiegend *FGFR1* (*FGFR1*-Amplifikationen in 23/243 = 9,5 % sowie *FGFR2*- und *FGFR3*-Amplifikationen in je 2/243 = 0,8 % Tumoren) [6]. Eine höhere *FGFR1*-Amplifikationsrate von 12,6–15 % konnte in weiteren Studien gefunden werden [24, 25]. Die starke Assoziation von *FGFR1*-Amplifikationen mit HPV-Negativität konnte in weiteren Arbeiten unabhängig bestätigt werden [5, 24, 26, 27]. Jedoch weisen nicht alle FGFR-amplifizierten Tumoren zwangsläufig auch eine FGFR-Überexpression auf, was beispielsweise über eine posttranskriptionelle Regulation der Expression über MicroRNA erklärt werden kann [28].

### Aktivierende Mutation

Aktivierende Mutationen der FGFR-Gene können zu einer Dysregulation der FGFR-Signalkaskade führen, indem diese Mutation zu einer Erhöhung der Kinase-Aktivität, zu einer ligandenunabhängigen Rezeptor-Dimerisierung oder einer veränderten Bindeaffinität gegenüber den Liganden führt [12]. Mutationen des FGFR-Signalwegs gehören zu den häufigsten Mutationen bei malignen Erkrankungen [29]. Innerhalb der 279 HNSCC-Tumoren des *The Cancer Genome Atlas* konnten FGFR-Mutationen in sieben Tumoren (2,5 %) nachgewiesen werden (*FGFR1*-Mutation in einem HPV-negativen Tumor, *FGFR2*-Mutationen in zwei HPV-negativen Tumoren und *FGFR3*-Mutationen in je zwei HPV-negativen und -positiven Tumoren) [6]. Höhere Mutationsraten von 14 % (*FGFR3*) und 4 % (*FGFR2*) wurden in einer weiteren Studie in HPV-positiven Tumoren gefunden, während HPV-negative Tumoren keine Mutationen trugen [5]. In Einzelfällen liegen auch bei papillären Schilddrüsenkarzinomen *FGFR3*-Mutationen vor [30]. Mutationen des FGFR-Signalwegs (beispielsweise *FGF16* und *FGFR4*) sind darüber hinaus bei adenoid-zystischen Speicheldrüsenkarzinomen zu finden [19].

### Genfusionen

Fusionsgene sind Hybride, die nach der strukturellen Umordnung von zwei zuvor voneinander unabhängigen Genen entstehen. Bisher konnten eine Reihe von Fusionspartnergenen von *FGFR1*–*3* in verschiedenen malignen Erkrankungen identifiziert werden, darunter *TACC1, TACC3 *(„transforming acidic coiled-coil containing protein 1“ und „transforming acidic coiled-coil containing protein 3“), *BAIAP2L1 *(„BAR/IMD domain containing adaptor protein 2 like 1“), *BICC1 *(„BicC family RNA binding protein 1“) und *AHCYL1 *(„adenosylhomocysteinase like 1“) [6, 31–37]. Entsprechend der jeweiligen Art der Genumlagerung kann die Aktivität der FGFR dadurch auf verschiedene Weise verändert werden: Die Kinasedomäne des Fusionsgenprodukts kann ligandenunabhängig konstitutiv aktiviert sein, oder das Gen kann in den regulatorischen Einfluss eines starken Promotors geraten, wodurch es zu einer Überexpression des Fusionsproteins kommt [31–33]. Bei HNSCC und Nasopharynxkarzinomen konnte die Fusion von *FGFR3* mit *TACC3* nachgewiesen werden [32, 37]. *FGFR3*-*TACC3*-Fusionen wurden bei zwei von 36 (6 %) HPV-positiven HNSCC-Tumoren des *The Cancer Genome Atlas* gefunden [6]. Die Fusion mit TACC3 führt zu einer konstitutiven FGFR3-Kinaseaktivität [31]. In Mausmodellen konnte die onkogene Eigenschaft des FGFR3-TACC3-Fusionsproteins für Tumoren des zentralen Nervensystems und der Lunge gezeigt werden [38]. Fusionen unter Beteiligung von *FGFR2* finden sich in ca. 0,5 % der papillären Schilddrüsenkarzinome [30].

### Veränderung der autokrinen/parakrinen Regulation

Veränderte autokrine oder parakrine Signalschleifen aufgrund einer verstärkten Freisetzung von FGF durch Tumorzellen oder dem Stroma können ebenfalls zu einer Dysregulation des FGFR-Signalwegs führen [12]. Die Amplifikation von FGF kann beispielsweise zu einer verstärkten FGF-Sekretion und veränderter autokriner Regulation führen [39, 40]. Eine besonders hohe Prävalenz von FGF-Amplifikationen bei HNSCC betrifft *FGF3* (22,9 %, 67/292), *FGF4* (21,2 %, 62/292) und *FGF19* (22,6 %, 66/292), welche in Chromosomenbande 11q13 zusammen mit dem Zellzyklusregulator Cyclin D1 (*CCND1*) lokalisiert sind [39]. Entsprechend einer Koamplifikation wurde eine *CCND1*-Amplifikation in 27 % (76/279) der HNSCC des *The Cancer Genome Altas* gefunden [6] und steht damit im Einklang mit der Prävalenz der *FGF3*-, *FGF4*- und *FGF5*-Amplifikation. Besonders häufig von diesen Koamplifikationen waren die HPV-negativen (31 %) im Vergleich zu den HPV-positiven Tumoren (3 %) betroffen [6].

### Alternatives Spleißen

Alternatives Spleißen der FGFR1–3 führt zur Expression verschiedener Isoformen mit unterschiedlicher Ligandenbindespezifität. Darüber hinaus können verkürzte Transkripte zur Bildung löslicher FGFR-Varianten führen [11].

## Inhibitoren des FGFR-Signalwegs

Für die zielgerichtete Inhibition des FGFR-Signalwegs werden in der Therapie von malignen Erkrankungen verschiedene Ansätze mittels TKI, mAb (mit und ohne Wirkstoff-Konjugat) oder einer Ligandenfalle verfolgt. Diese Wirkstoffe befinden sich aktuell größtenteils in der präklinischen oder klinischen Prüfung.

### TKI

TKI sind kleine Moleküle, die in der Lage sind, die Kinaseaktivität der RTK über verschiedene Wirkmechanismen zu hemmen [9, 41]. TKI wirken kompetitiv zur Adenosintriphosphat (ATP)-Bindung an die hochkonservierte ATP-Bindestelle der RTK oder kompetitiv zur Bindung des Substrats der RTK. Andere TKI inhibieren kompetitiv die phosphorylierende RTK-Domäne, oder sie binden außerhalb des aktiven Zentrums und bewirken über eine Konformationsänderung eine nichtkompetitive (allosterische) Hemmung. Entsprechend der jeweiligen Affinität zu verschiedenen RTK-spezifischen Bindungsstellen sind TKI mehr oder weniger selektiv für bestimmte RTK. Aufgrund der hohen Konservierung der ATP-Bindestelle der RTK wirken TKI, die mit der ATP-Bindestelle konkurrieren, nichtselektiv hemmend auf verschiedene RTK, beispielsweise VEGFR („vascular endothelial growth factors“), PDGFR („platelet-derived growth factor receptors“), FGFR, aber auch FLT3 („fms related receptor tyrosine kinase 3“), RET („ret proto-oncogene“) und KIT („KIT proto-oncogene, receptor tyrosine kinase“). Diese nichtselektiven TKI können aufgrund der Bandbreite der Zielmoleküle eine höhere Wirkung, aber auch stärkere Nebenwirkungen aufweisen. Sie werden vornehmlich als Angiogenesehemmer eingesetzt. Entsprechend ist für die Behandlung von Tumoren mit dysreguliertem FGFR-Signalweg die Verwendung von selektiven, hochpotenten FGFR-TKI vielversprechender [9, 12]. Eine Vielzahl verschiedener FGFR-TKI befinden sich zurzeit in unterschiedlichen Stadien der klinischen Entwicklung und Prüfung (Tab. 1; [9, 12, 13, 42, 43]). Der selektive TKI Erdafitinib hat kürzlich die globale Zulassung durch die US-amerikanische Gesundheitsbehörde FDA (Food and Drug Administration) für die Behandlung von metastasierten Urothelkarzinomen mit *FGFR3*-Mutationen oder *FGFR2*/*3*-Genfusionen erhalten [44]. Weitere selektive FGFR-TKI befinden sich in klinischer Prüfung der Phase III (AZD4547 [AstraZeneca PLC, London, UK], Futibatinib [Taiho Pharmaceutical, Tokio, Japan], Infigratinib [QED Therapeutics, Inc., San Francisco, CA, USA], Rogaratinib [Bayer AG]). Nichtselektive TKI (Anlotinib [Advenchen Laboratories, LLC., Moorpark, CA, USA und Chia Tai-Tianqing Pharmaceutical Holdings Co., Ltd., Jiangsu, China], Nintedanib [Boehringer Ingelheim AG & Co. KG, Ingelheim am Rhein, Deutschland], Ponatinib [Ariad Pharmaceuticals, Inc., Cambridge, MA, USA]) sind für die Behandlung von Leukämien oder als Angiogenesehemmer für nichtkleinzellige Lungenkarzinome regional zugelassen [45, 46].

**Table 1:** 

Wirkstoff	Typ	Ziel	Hersteller	Entwicklungsstand	Klinische Prüfung HNSCC	Weiterer HNO-Bezug
Aprutumab-Ixadotin (BAY1187982)	Antikörper-Wirkstoff-Konjugat	FGFR2	Bayer AG (Leverkusen, Deutschland)	Phase I, abgebrochen	*N* = 1, Phase I (NCT02368951^f^)	Adenoid-zystische Karzinome der Parotis und der Zunge (je *N* = 1, NCT02368951^f^)
LY3076226	Antikörper-Wirkstoff-Konjugat	FGFR3	Eli Lilly and Company (Indianapolis, IN, USA), ImmunoGen, Inc. (Waltham, MA, USA)	Phase I, abgebrochen	Nein	Nein
FP-1039 (GSK3052230)	FGF-Ligandenfalle	FGF	Five Prime Therapeutics, Inc. (South San Francisco, CA, USA)	Phase II, abgebrochen	*N* = 4, Phase I (NCT00687505^e^)	Nein
Aprutumab (BAY1179470)	mAb	FGFR2	Bayer AG	Phase I, abgebrochen	Nein	Nein
Bemarituzumab (FPA144)	mAb	FGFR2b	Five Prime Therapeutics, Inc.	Phase III (Magenkarzinom), laufend	Nein	Nein
Vofatamab (B-701)	mAb	FGFR3	Rainier Therapeutics, Inc. (San Leandro, CA, USA)	Phase I/II, abgebrochen	Nein	Nein
Anlotinib (AL3818)	TKI (nichtselektiv)	FGFR1–4	Chia Tai-Tianqing Pharmaceutical Holdings Co., Ltd., Advenchen Laboratories, LLC.	Zugelassen (NSCLC; China)	*N* = NV, Phase II (NCT04203719^b^)	Kopf-Hals-Adenokarzinome, Phase II (*N* = 21, NCT03591666^e^); Nasopharynxkarzinom, Phase III, Phase I/II (*N* = 336, NCT03601975^a^,* N* = 40, NCT03639467^a^); Schilddrüse, Phase I (*N* = 1, NCT01833923^e^)
Brivanib-Alaninat (BMS-582664), Brivanib (BMS-540215)	TKI (nichtselektiv)	FGFR1–3	Bristol-Myers Squibb Co. (New York City, NY, USA)	Phase III (HCC, CRC), fehlgeschlagen; Phase II, laufend	Nein	Nein
Danusertib (PHA-739358)	TKI (nichtselektiv)	FGFR1	Nerviano Medical Sciences S.r.l. (Nerviano, Italien)	Phase II, abgebrochen	Nein	Nein
Dovitinib (TKI258)	TKI (nichtselektiv)	FGFR1/3	Novartis AG, Oncology Venture A/S (Hørsholm, Dänemark)	Phase III (RCC), fehlgeschlagen; Biomarker-Evaluation laufend	Nein	Schilddrüse, Phase II (*N* = 40, NCT01964144^e^); adenoid-zystische Karzinome, Phase II (*N* = 35, NCT01524692^e^; *N* = 21, NCT01678105^e^, *N* = 32, NCT01417143^e^)
Lucitanib (E-3810)	TKI (nichtselektiv)	FGFR1–2	Clovis Oncology, Inc. (Boulder, CO, USA)	Phase II, laufend	Nein	Schilddrüse, Phase I/II (*N* = 9, NCT01283945^e^)
Masitinib (Masivet®, AB1010)	TKI (nichtselektiv)	FGFR3	AB Science (Paris, Frankreich)	Phase III (Adenokarzinom der Prostata und des Pankreas), laufend	Nein	Nein
MAX-40279	TKI (nichtselektiv)	FGFR^h^	MaxiNovel Pharmaceuticals, Inc. (Guangzhou, China)	Phase I, laufend	Nein	Nein
Nintedanib (Vargatef®, BIBF1120)	TKI (nichtselektiv)	FGFR1–3	Boehringer Ingelheim AG & Co. KG	Zugelassen (NSCLC/Adenokarzinom; EU)	*N* = NV, Phase II (NCT03292250^a^)	Speicheldrüse, Phase II (*N* = 20, NCT02558387^e^); Schilddrüse, Phase II (*N* = 45; NCT01788982^e^)
Orantinib (TSU-68, SU-6668)	TKI (nichtselektiv)	FGFR1	Pifzer, Inc. (New York City, NY, USA), Taiho Pharmaceutical	Phase III (HCC), abgebrochen	Nein	Nein
Ponatinib (AP24534)	TKI (nichtselektiv)	FGFR1–3	Ariad Pharmaceuticals, Inc.	Zugelassen (CML, AML; USA)	*N* = NV, Phase II/III (NCT01761747^f^)	Schilddrüse, Phase II (*N* = 31, NCT03838692^b^;* N* = 3, NCT01838642^f^)
Surufatinib (Sulfatinib, HMPL-012)	TKI (nichtselektiv)	FGFR1	Hutchison MediPharma Ltd. (Shanghai, China)	Phase II, laufend	Nein	Schilddrüse, Phase II (*N* = 18, NCT02614495^e^)
XL228	TKI (nichtselektiv)	FGFR1–3	Exelixis Inc. (Alameda, CA, USA)	Phase I, abgebrochen	Nein	Nein
XL999	TKI (nichtselektiv)	FGFR1/3	Symphony Evolution, Inc. (Rockville, MD, USA)	Phase II, abgebrochen	Nein	Nein
Alofanib (RPT835)	TKI (selektiv)	FGFR2	Ruspharmtech (St. Petersburg, Russland)	Phase I, laufend	Nein	Nein
ASP5878	TKI (selektiv)	FGFR1–4	Astellas Pharma, Inc. (Tokio, Japan)	Phase I, beendet	Nein	Nein
AZD4547	TKI (selektiv)	FGFR1–4	AstraZeneca PLC	Phase II/III (NSCLC/squamös), beendet	Nein	Nein
Debio 1347, CH5183284	TKI (selektiv)	FGFR1–3	Debiopharm (Lausanne, Schweiz)	Phase I/II, laufend	Möglich, Phase II (NCT03834220^c^)	Möglich, Phase II (NCT03834220^c^)
Derazantinib (ARQ 087)	TKI (selektiv)	FGFR1–3	Basilea Pharmaceutica AG (Basel, Schweiz)	Phase II, laufend	Nein	Nein
E7090	TKI (selektiv)	FGFR1–3	Eisai Co., Ltd. (Tokio, Japan)	Phase I, laufend	Nein	Nein
Erdafitinib (Balversa®, JNJ-42756493)	TKI (selektiv)	FGFR1–4	Janssen Pharmaceutica N.V.	Zugelassen (Urothelkarzinom, USA)	*N* ≤ 11, Phase I (NCT01703481^e^); möglich, Phase I/II (NCT04083976^a^, NCT03547037^a^, NCT03210714^a^)	Möglich, Phase I/II (NCT04083976^a^, NCT03547037^a^, NCT03210714^a^)
FGF401	TKI (selektiv)	FGFR4	Novartis AG	Phase I, beendet	Nein	Nein
Fisogatinib (BLU-554)	TKI (selektiv)	FGFR4	Blueprint Medicines Corp. (Cambridge, MA, USA)	Phase I, laufend	Nein	Nein
Futibatinib (TAS-120)	TKI (selektiv)	FGFR1–4	Taiho Pharmaceutical	Phase III (CCC), laufend	Möglich, Phase II (NCT04189445^b^)	Möglich, Phase II (NCT04189445^b^)
H3B-6527	TKI (selektiv)	FGFR4	H3 Biomedicine, Inc. (Cambridge, MA, USA), Eisai Co., Ltd.	Phase I, laufend	Nein	Nein
HMPL-453	TKI (selektiv)	FGFR1–3	Hutchison MediPharma Ltd.	Phase I/II, laufend	Möglich, Phase I/II (NCT03160833^a^)	Möglich, Phase I/II (NCT03160833^a^)
INCB062079	TKI (selektiv)	FGFR4	Incyte, Inc. (Wilmington, DE, USA)	Phase I, laufend	Möglich, Phase I (NCT03144661^a^)	Nasopharynxkarzinom, Phase I (NCT03144661^a^)
Infigratinib (BGJ398)	TKI (selektiv)	FGFR1–4	QED Therapeutics	Phase III (CCC), laufend	Je *N* = 1, Phase I (NCT02706691^g^, NCT01928459^e^)	Nein
LY2874455	TKI (selektiv)	FGFR1–4	Eli Lilly and Company	Phase I, laufend	Nein	Nein
Pemigatinib (INCB054828)	TKI (selektiv)	FGFR1–3	Incyte, Inc.	Phase II, laufend	Möglich, Phase I/II (NCT04003623^a^, NCT03235570^a^, NCT02393248^a^, NCT03822117^a^)	Möglich, Phase I/II (NCT04003623^a^, NCT03235570^a^, NCT02393248^a^, NCT03822117^a^)
PRN1371	TKI (selektiv)	FGFR1–4	Principia Biopharma, Inc. (South San Francisco, CA, USA)	Phase I, laufend	Nein	Nein
Rogaratinib (BAY1163877)	TKI (selektiv)	FGFR1–4	Bayer AG	Phase II/III (Urothelkarzinom), laufend	*N* = 8, Phase I, (NCT01976741^e^); *N* = NV, Phase I (NCT02592785^e^); möglich, Phase I (NCT03788603^a^, NCT03517956^a^)	Möglich, Phase I (NCT03788603^a^, NCT03517956^a^)
CPL304110	TKI^h^	FGFR^h^	Celon Pharma S.A. (Kiełpin, Polen)	Phase I, laufend	Nein	Nein

*AML* Akute Myeloische Leukämie, *CCC* „cholangiocellular carcinoma“, Cholangiozelluläres Karzinom, Gallengangskarzinom, *CRC* „colorectal cancer“, Kolorektalkarzinom, *HCC* „hepatocellular carcinoma“, Hepatozelluläres Karzinom, *NSCLC* „non-small cell lung cancer“, Nichtkleinzelliges Lungenkarzinom, *RCC* „renal cell carcinoma“, Nierenzellkarzinom, *NV* nicht verfügbar

^a^Rekrutierend

^b^Noch nicht rekrutierend

^c^Laufend, nicht rekrutierend

^d^Abgeschlossen, ohne Ergebnisse

^e^Abgeschlossen, mit Ergebnissen

^f^Abgebrochen

^g^Abgebrochen wegen geringer Rekrutierung

^h^Nicht näher spezifiziert

### mAb

Gegen FGFR gerichtete therapeutische monoklonale Antikörper inhibieren die FGFR-Signalkaskade über eine Blockade der Ligandenbindung oder eine Dimerisierung. Diese mAb sind spezifisch für bestimmte FGFR-Isoformen und können daher ein günstigeres Nebenwirkungsspektrum aufweisen. FGFR-mAb können darüber hinaus als Konjugate mit Toxinen angewendet werden.

### Ligandenfallen

Ligandenfallen binden und neutralisieren selektiv die an FGFR1-bindenden FGF und verhindern so die Aktivierung von FGFR1 [47, 48]. Der lösliche Wirkstoff FP-1039 stellt im Wesentliche ein Fusionsprotein der extrazellulären ligandenbindenden Domäne von FGFR1 mit dem Immunglobulin G1 dar [47, 48].

## Präklinische und klinische Wirksamkeit und Verträglichkeit von gegen FGFR-gerichteten Wirkstoffen

Die präklinische und klinische Wirksamkeit sowie die Verträglichkeit von gegen FGFR-gerichteten Wirkstoffen sind vom spezifischen Wirkstoff, von der Tumorentität und von den molekularen Veränderungen des individuellen Tumors abhängig [12].

### Wirksamkeit und Verträglichkeit von TKI

In-vitro-Untersuchungen von 58 Lungenkarzinom-Zelllinien ergaben, dass die FGFR1-mRNA- und -Proteinexpression, nicht jedoch die Genamplifikation prädiktiv für die Sensitivität gegenüber dem FGFR-TKI Ponatinib ist [49]. Dieses Ergebnis konnte für HNSCC-Zelllinien, die mit dem FGRF-TKI Infigratinib behandelt wurden, bestätigt werden [50]. Die Sensitivität von HNSCC-Zelllinien gegenüber Infigratinib konnte darüber hinaus für FGFR3-mRNA-exprimierende Linien bestätigt werden [51]. Noch sind Erfahrungen bezüglich der Wirksamkeit von TKI bei Patienten mit metastasiertem HNSCC und weiteren Karzinomen des Kopf-Hals-Bereichs rar (Tab. 2). Klinische Daten einer Phase-I-Studie (Patienten mit metastasierten soliden Tumoren, inklusive sieben HNSCC-Patienten; NCT01976741) zeigten, dass nur 15 % (15/100) der Patienten mit FGFR-überexprimierenden Tumoren auf eine Behandlung mit dem FGFR-Inhibitor Rogaratinib ansprachen. Eine Subgruppenanalyse zeigte jedoch eine besonders hohe Ansprechrate mit 67 % (10/15) in FGFR-überexprimierenden Tumoren ohne detektierte genomische FGFR-Veränderung [52]. Bei einem der sieben evaluierbaren HNSCC-Patienten konnte ein partielles Ansprechen und bei zwei Patienten eine Stabilisierung der Tumorerkrankung erreicht werden, während bei vier Patienten ein Progress auftrat [52]. Eine frühere Auswertung dieser Studie zusammen mit einer weiteren Phase-I-Studie (NCT02592785) ergab unter den insgesamt zehn eingeschlossenen und evaluierbaren HNSCC-Patienten ein partielles Ansprechen bei einem Patienten und bei sechs Patienten eine Stabilisierung der Erkrankung, während bei zwei Patienten ein Progress eintrat [52, 53]. Tumoren von sechs der zehn Patienten wiesen eine FGFR3-mRNA-Überexpression auf, darunter die beiden Patienten mit partiellem Ansprechen [52, 53]. Seit 2017 läuft die biomarkergesteuerte Phase-II-Umbrella-Studie der *European Organisation for Research and Treatment of Cancer* (EORTC, NCT03088059) [54] mit dem Ziel, eine individuelle Therapie, basierend auf den spezifischen molekularen Eigenschaften des HNSCC, anzupassen. In dieser Studie erhalten HNSCC-Patienten mit FGFR1/2/3-mRNA-Überexpression eine Therapie mit Rogaratinib. Ergebnisse dieser Studie werden Ende 2021/Anfang 2022 erwartet.

**Table 2:** 

Tumor	Wirkstoff/Wirkstoffklasse	Patientenzahl	Klinischen Wirksamkeit	Studie	Molekulare Veränderungen	Referenz
HNO (adenoid-zystisch)	Dovitinib/TKI (nichtselektiv)	*N* ≤ 34	70,6 % DCR, 5,9 % ORR	NCT01524692 (Phase II)	Keine Angabe	Dillon et al. [66]
HNO (Adenokarzinome)	Anlotinib/TKI (nichtselektiv)	*N* = 21	81,0 % DCR, 19,1 % ORR	NCT03591666 (Phase II)	Keine Angabe	Jiang et al. [64]
HNO (nicht weiter spezifiziert)	Erdafitinib/TKI (selektiv)	*N* = 11	Keine Angabe	NCT01703481 (Phase I)	Keine Angabe	Bahleda et al. [55]
HNO (nicht weiter spezifiziert)	Infigratinib/TKI (selektiv)	*N* = 1	100 % (1/1) PR	NCT01928459 (Phase I)	Keine Angabe	Hyman et al. [59]
HNSCC	Rogaratinib/TKI (selektiv)	*N* = 7	14,3 % (1/7) PR, 28,6 % (2/7) SD, 57,1 % (4/7) PD	NCT01976741 (Phase I)	Vorwiegend FGFR-überexprimierend ohne genomische Veränderungen	Schuler et al. [52]^a^
HNSCC	Rogaratinib/TKI (selektiv)	*N* = 10	10 % (1/10) PR, 60 % (6/10) SD, 20 % (2/10) PD, 10 % (1/10) unbekannt	NCT02592785 (Phase I), NCT01976741 (Phase I)	Vorwiegend FGFR3-überexprimierend ohne genomische Veränderungen	Joerger et al. [53]^a^
HNSCC	Keine Angabe/TKI (selektiv)	*N* = 1	100 % (1/1) CR	NCT02152254	*FGF3, FGF4, FGF19, FGF6* und *FGF3* Amplifikation	Dumbrava et al. [60]
HNSCC, Schilddrüsenkarzinom	Anlotinib/TKI (nichtselektiv)	*N* ≤ 140	Noch nicht bekannt	NCT04203719 (Phase II)	Keine Angabe	–
Schilddrüsenkarzinom	Anlotinib/TKI (nichtselektiv)	*N* = 1	100 % (1/1) SD	NCT01833923 (Phase I)	Keine Angabe	Sun et al. [63]
Schilddrüsenkarzinom	Dovitinib/TKI (nichtselektiv)	*N* = 40	69,1 % DCR, 20,5 % ORR	NCT01964144 (Phase II)	Keine Angabe	Lim et al. [65]
Schilddrüsenkarzinom	Lucitanib/TKI (nichtselektiv)	*N* = 9	44,4 % ORR, 22,0 % (2/9) CR, 22,0 % (2/9) PR	NCT01283945 (Phase I/II)	Keine Angabe	Soria et al. [68]
Schilddrüsenkarzinom	Nintedanib/TKI (nichtselektiv)	*N* = 70	3,71 (TKI) vs 2,86 (Placebo) Monate PFS	NCT01788982 (Phase II)	Keine Angabe	Schlumberger et al. [69]
Schilddrüsenkarzinom	Surufatinib/TKI (nichtselektiv)	*N* = 18	23,5 % (4/17) PR	NCT02614495 (Phase II)	Keine Angabe	Chen et al. [71]
Speicheldrüsenkarzinom (adenoid-zystisch)	Dovitinib/TKI (nichtselektiv)	*N* = 21	71,4 % DCR	NCT01678105 (Phase II)	Keine Angabe	Hotte et al. [67]
Speicheldrüsenkarzinom (ohne Angabe der Histologie)	Nintedanib/TKI (nichtselektiv)	*N* = 20	75 % DCR, 0 % ORR	NCT02558387 (Phase II)	Keine Angabe	Kim et al. [70]

*ORR* „objective response rate“, *DCR* „disease control rate“, *PR* „partial response“, *CR* „complete response“, *SD* „stable disease“, *PD* „progressive disease“, *PFS* „progression-free survival“

^a^Studienüberlappend

In eine Phase-I-Studie (NCT01703481) mit dem selektiven FGFR-TKI Erdafitinib wurden 187 Patienten mit verschiedenen fortgeschrittenen soliden Tumoren eingeschlossen und eine objektive Ansprechrate von 11 % (21/187) erreicht [55]. Bei weiteren 16 % (29/187) konnte eine Stabilisierung der Tumorerkrankung erzielt werden. Besonders hoch war die Ansprechraten bei Urothel- (46 %) und Cholangiokarzinomen (27 %) mit FGFR-Mutationen oder -Fusionen [55]. Bei 6 % (11/187) der eingeschlossenen Patienten handelte es sich um Patienten mit Kopf-Hals-Tumoren ohne genauere Angabe der Histologie, über deren Ansprechen auf die Therapie jedoch nicht gesondert berichtet wurde. Eine kürzlich veröffentlichte Phase-II-Studie (NCT02365597) zeigte in vorbehandelten Patienten mit fortgeschrittenem inoperablen oder metastasiertem Urothelkarzinom mit einer *FGFR3*-Mutation oder *FGFR2*/*3*-Genfusion eine Ansprechrate von 40 % (dabei 3 % Komplettremissionen) nach Behandlung mit Erdafitinib [7]. Aufgrund dieser Studienergebnisse erhielt Erdafitinib in dieser Anwendung eine beschleunigte Zulassung durch die FDA. In der Studie zeigten 17 der 99 (17 %) eingeschlossenen Patienten *FGFR3*-*TACC3*-Fusionen im Tumor [7]. Eine Ansprechrate von 25,4 % und eine Stabilisierung der Erkrankung bei weiteren 38,8 % der 67 inkludierten Patienten mit metastasiertem Urothelkarzinom, die *FGFR3*-Mutationen oder -Fusionen trugen, konnte in der Expansionskohorte einer Phase-I-Studie des TKI Infigratinib erreicht werden [56]. In dieser Studie trugen 7,5 % der Tumoren *FGFR3*-*TACC3*-Fusionen. Über das Ansprechen in der Subgruppe der Tumoren mit *FGFR3*-*TACC3*-Fusionen wurde nicht gesondert berichtet [7]. Allerdings zeigen präklinische In-vitro- und In-vivo-Daten, dass *FGFR3*-*TACC3*-positive Tumoren sensitiv für FGFR-Inhibitoren sind [31, 32, 57]. Die prädiktive Eigenschaft von FGFR-Fusionen für das Ansprechen auf FGFR-TKI konnte auch in einer Phase-II-Studie (NCT02150967) des Wirkstoffs Infigratinib bei Patienten mit vorbehandeltem Cholangiokarzinom gezeigt werden [58]. In dieser Studie sprachen 18,8 % (9/48) der Patienten, deren Tumoren eine *FGFR2*-Fusion trugen, auf die FGFR-Inhibierung an [58]. In den präklinischen Studien wurden allerdings bisher keine HNSCC-Zelllinien hinsichtlich der Sensitivität gegenüber einer FGFR-Inhibition getestet. Da *FGFR3*-*TACC3*-Fusionen vereinzelt bei HPV-positiven HNSCC-Tumoren gefunden werden [6], könnte eine FGFR-gerichtete Therapie bei diesen Patienten ein vielversprechender Ansatz sein. Für einen Patienten mit einem Kopf-Hals-Tumor unbeschriebener Histologie konnte anekdotisch ein partielles Ansprechen auf Infigratinib gefunden werden (NCT01928459) [59]. Eine geplante Studie zu Infigratinib in HNSCC (NCT02706691) wurde wegen ungenügender Patientenrekrutierung allerdings beendet.

Eine Kasuistik beschreibt eine Komplettremission bei einem Patienten mit metastasiertem HNSCC unter Behandlung mit einem nicht näher beschriebenen selektiven FGFR-Inhibitor im Rahmen der IMPACT-II-Studie (NCT02152254). Der Tumor mit unbekanntem HPV-Status wies Amplifikationen von *FGF3, FGF4, FGF19, FGF6* und *FGF3* auf [60].

Wie die Wirksamkeit, so ist auch die Verträglichkeit der TKI vom jeweiligen Wirkstoff abhängig. Generell führen selektive TKI zu geringeren Nebenwirkungen im Vergleich zu nichtselektiven TKI [12]. In der Zulassungsstudie (NCT02365597) von Erdafitinib zur Behandlung des lokal fortgeschrittenen oder metastasierten Urothelkarzinoms kam es bei 46 % der Patienten zu Nebenwirkungen vom Grad 3–4 (v. a. Hyponatriämie, Stomatitis und Asthenie). Dreizehn der 99 (13 %) behandelten Patienten brachen die Therapie wegen der Nebenwirkungen ab. Es gab keine behandlungsbezogenen Todesfälle [7]. In der Phase-I-Dosisfindungsstudie (NCT01976741) von Rogaratinib bei Patienten mit fortgeschrittenen Tumoren wurden 126 Patienten behandelt. Bei 36 von 126 (29 %) kam es zu Nebenwirkungen vom Grad 3–4 (v. a. Müdigkeit und asymptomatisch erhöhte Lipase). Sechs der 126 (5 %) behandelten Patienten brachen die Therapie wegen der Nebenwirkungen ab. Es gab ebenfalls keine behandlungsbezogenen Todesfälle [52].

Wirksamkeit und Verträglichkeit von TKI konnte ebenfalls für die Behandlungen weiterer maligner Erkrankungen des Kopf-Hals-Bereichs gezeigt werden. Der nichtselektive TKI Anlotinib inhibiert VEGFR, PDGFR, FGFR und KIT [61, 62]. In einer Phase-I-Studie (NCT01833923) war ein Patient mit einem Schilddrüsenkarzinom eingeschlossen, bei dem eine Stabilisierung der Erkrankung erreicht werden konnte [63]. In die folgende Phase-II-Studie wurden 21 Patienten mit Adenokarzinomen des Kopf-Hals-Bereichs eingeschlossen (NCT03591666) [64]. Die häufigsten Nebenwirkungen ≥Grad 3 waren arterielle Hypertonie (28,6 %) und Hand-Fuß-Syndrom (9,5 %). Bei 19,1 % der Patienten wurde ein objektives Ansprechen beobachtet, und es konnte eine Erkrankungskontrollrate von 81,0 % erreicht werden [64]. Eine Phase-II-Studie (NCT04203719) mit einem geplanten Einschluss von 140 Patienten mit Plattenepithelkarzinomen des Kopf-Hals-Bereichs, der Lunge sowie Schilddrüsenkarzinomen und Pleuramesotheliomen läuft zurzeit in China an.

Dovitinib ist ein gegen FGFR, VEGFR, PDGFR, KIT und FLT3 gerichteter TKI. In einer Phase-II-Studie (NCT01964144) mit 40 eingeschlossenen Schilddrüsenkarzinom-Patienten (23 papilläre, 12 medulläre und fünf follikuläre Schilddrüsenkarzinome) zeigte sich eine mäßige Wirkung bei handhabbaren Nebenwirkungen. Bei den 39 evaluierbaren Patienten lag die Ansprechrate bei 20,5 % (8/39) und die Erkrankungskontrollrate bei 69,1 % (26/39). Die häufigsten Nebenwirkungen waren Grad 1 und 2 und umfassten Diarrhö (53,8 %), Anorexie (35,8 %), Übergeben (25,6 %), Müdigkeit (23,1 %) und Übelkeit (20,5 %) [65]. In eine weitere Phase-II-Studie (NCT01524692) wurden 34 Patienten mit adenoid-zystischen Karzinomen vorwiegend des Kopf-Hals-Bereichs eingeschlossen und eine vergleichbare Erkrankungskontrollrate von 70,6 % (24/34) bei 5,9 % (2/34) partiell ansprechenden Erkrankungen gefunden [66]. Vergleichbare Ergebnisse lieferte eine zweite Phase-II-Studie (NCT01678105) mit 21 Patienten mit adenoid-zystischen Karzinomen der Speicheldrüse. Hier konnte eine Stabilisierung bei 71,4 % (15/21) der Probanden erreicht werden [67]. Nachdem der klinische Endpunkt einer Phase-III-Studie im Nierenzellkarzinom nicht erreicht werden konnte, wurde die Entwicklung des Wirkstoffs durch Novartis zunächst ausgesetzt. Zwischenzeitlich prüft die Firma Oncology Venture A/S eine relative Erhöhung der Wirksamkeit durch eine biomarkerbasierte Stratifizierung des Patientenkollektivs.

In einer Phase-I/II-Studie (NCT01283945) des gegen VGFR1–3, FGFR1/2 und PDGFR gerichteten TKI Lucitanib wurden unter anderem neun Patienten mit Schilddrüsenkarzinomen eingeschlossen. Bei handhabbaren Nebenwirkungen konnte bei 44,4 % (4/9) ein Ansprechen (je zwei Komplettremissionen und partielle Remissionen) beobachtet werden [68].

In zwei Phase-II-Studien wurde der in Europa für die Behandlung des Adenokarzinoms der Lunge zugelassene, gegen VGEFR1–3, FGFR1–3 und PDGFR gerichtete TKI Nintedanib an Patienten mit Schilddrüsen- (NCT01788982) und Speicheldrüsentumoren (NCT02558387) getestet. Eine Zweitlinientherapie der Schilddrüsenkarzinome mit Nintedanib führte im Vergleich zu Placebo zu einer leichten Verlängerung des progressionsfreien Überlebens (3,71 im Vergleich zu 2,86 Monate, *p* = 0,095) [69]. Nintedanib bei Patienten mit rekurrenten oder metastasierten Speicheldrüsentumoren führte zwar bei 75 % (15/20) der Patienten zu einer Stabilisierung der Erkrankung, allerdings war kein objektives Ansprechen zu beobachten, sodass die Studie gestoppt wurde [70].

Der unter anderem gegen FGFR1 gerichtete nichtselektive TKI Surufatinib wurde in einer Phase-II-Studie (NCT02614495) an 18 Patienten mit Schilddrüsenkarzinomen getestet. Bei 17 evaluierbaren Patienten lag die Ansprechrate (partielles Ansprechen) bei 23,5 % (4/17). Die häufigsten Nebenwirkungen waren Proteinurie (72,2 %; Grad 3–4: 22,2 %), Hypertriglyzeridämie (50,0 %; Grad 3–4: 0 %), arterielle Hypertonie (44,4 %; Grad 3–4: 16,7 %), Bilirubin-Anstieg im Blut (44,4 %; Grad 3–4: 5,6 %) und Diarrhö (33,3 %; Grad 3–4: 0 %) [71].

### Wirksamkeit und Verträglichkeit der Antikörper-Wirkstoff-Konjugate

Aprutumab-Ixadotin ist ein gegen FGFR2 gerichtetes Antikörper-Wirkstoff-Konjugat, dass in vivo und in vitro geeignete Eigenschaften zeigte, sodass eine Phase-I-Studie initiiert wurde (NCT02368951) [72, 73]. Eine Zwischenanalyse nach Einschluss von 20 Patienten ergab jedoch eine geringe Verträglichkeit. Die häufigsten Nebenwirkungen ≥Grad 3 waren Anämie, erhöhte Aspartat-Aminotransferase, Proteinurie und Thrombopenie. Fünf Patienten (darunter ein Patient mit Mundbodenkarzinom) verließen die Studie aufgrund von Nebenwirkungen. Bei 14 der verbleibenden 15 Patienten entwickelt sich ein Progress, darunter ein Patient mit adenoid-zystischem Parotiskarzinom. Bei einem Patienten mit adenoid-zystischem Karzinom der Zunge kam es zunächst zu einer Stabilisierung der Erkrankung [73]. Die Entwicklung von Aprutumab-Ixadotin wurde in Anbetracht des ungünstigen Nutzen-Risiko-Profils beendet.

In-vivo-Studien des gegen FGFR3 gerichteten Antikörper-Wirkstoff-Konjugats LY3076226 zeigten eine robuste Antitumoraktivität bei FGFR3-mutierten/fusionierten Urothelkarzinomzelllinien [74]. Eine Phase-I-Studie (NCT02529553) mit 37 Patienten mit FGFR3-mutierten/fusionierten Tumoren, darunter entsprechend den Einschlusskriterien möglicherweise auch HNSCC-Patienten, wurde 2018 abgeschlossen. Die Veröffentlichung der Ergebnisse ist zwar noch ausstehend, jedoch wurde die Entwicklung von LY3076226 zwischenzeitlich mutmaßlich eingestellt.

### Wirksamkeit und Verträglichkeit der mAb

In präklinischen Studien des Anti-FGFR2-mAb Aprutumab traten keine toxikologisch relevanten Auffälligkeiten auf. Erste Hinweise auf Wirksamkeit konnten in Xenograft-Modellen mit FGFR2-exprimierenden Zelllinien gefunden werden [75–77]. Basierend auf diesen Ergebnissen wurde eine Phase-I-Studie (NCT01881217) mit 35 Patienten unter Fokus auf FGFR2-exprimierende Magenkarzinome durchgeführt. Ergebnisse dieser Studie sind bisher nicht publiziert, was auf eine fehlende Wirksamkeit zurückzuführen sein könnte, wie dies schon für das entsprechende Antikörper-Wirkstoff-Konjugat Aprutumab-Ixadotin der Fall war [73].

Bemarituzumab ist ein gegen FGFR2b gerichteter mAb, der in einer Phase-I-Studie (NCT02318329) mit 64 Patienten mit verschiedenen soliden Tumoren (23 Patienten) und 41 Patienten mit gastroösophagealen Adenokarzinomen getestet wurde [78–80]. Dosislimitierende Toxizität wurde nicht beobachtet [78]. Die häufigsten Nebenwirkungen umfassten verminderten Appetit (32,8 %), Müdigkeit (25,0 %), Übelkeit (23,4 %), Übergeben und Anämie (jeweils 20,3 %), trockene Augen (15,8 %), Diarrhö (14,1 %), Hypoalbuminämie, Fieber und Gewichtsverlust (je 12,5 %) sowie Verstopfung, Dehydrierung, periphere Ödeme und eine erhöhte Aspartat-Aminotransferase (jeweils 10,9 %) [80]. Dreißig der 64 Patienten (46,9 %) erlitten mindestens eine Nebenwirkung vom Grad 3 [80]. Die Wirksamkeit wurde für 21 Patienten mit gastroösophagealen Adenokarzinomen mit hoher FGFR2b-Expression beschrieben. Die objektive Ansprechrate lag bei 19,0 % (darunter vier Patienten mit partiellem Ansprechen) und einer Erkrankungskontrollrate von 55,6 % [80]. Für die anderen Tumorentitäten wurde die Beschreibung der Wirksamkeit bisher nicht veröffentlicht. Aktuell läuft eine Phase-III-Studie (NCT03343301) mit FGFR2b-selektierten gastroösophagealen Adenokarzinomen [80, 81].

Vofatamab ist ein gegen FGFR3 gerichteter mAb, der als Monotherapie und in Kombination mit Docetaxel oder Pembrolizumab bei Patienten mit Urothelkarzinomen getestet wurde (NCT02401542, NCT03123055, NCT02925533). Vofatamab allein und in Kombination mit Docetaxel zeigte sich als gut verträglich mit wenigen Nebenwirkungen vom Grad 3 [82]. Sieben von 55 (12,7 %) Patienten sprachen objektiv auf die Vofatamab-Monotherapie oder -Kombinationstherapie mit Docetaxel an [82]. Bei einem Patienten mit *FGFR3*-mutierten/fusioniertem Tumor wurde eine Komplettremission erreicht [83]. Bei 30 % der Urothelkarzinom-Patienten mit *FGFR3*-wildtypischen Tumoren wurde ein Ansprechen auf die Kombinationstherapie mit Pembrolizumab erreicht (NCT03123055) [84]. Dieses Ansprechen erscheint höher als das Ansprechen, das bei einer Pembrolizumab-Monotherapie zu erwarten wäre. Zwischenzeitlich wurde die zweite Studie von Vofatamab plus Pembrolizumab (NCT02925533) wegen Sicherheitsbedenken jedoch abgebrochen.

### Wirksamkeit und Verträglichkeit der Ligandenfalle

Die Ligandenfalle FP-1039 bindet aufgrund des Wirkmechanismus keine endokrin wirkenden FGF (FGF19, FGF21 und FGF23), da diese einen zellmembranständigen Kofaktor (Klotho) für die Bindung benötigen. Dies suggeriert eine geringere Toxizität im Vergleich zu anderen FGFR-Signalweg-Inhibitoren [48]. Entsprechend konnte in zwei Phase-I-Studien (NCT01868022, NCT00687505) eine gute Verträglichkeit gezeigt werden, ohne dass für FGFR-Inhibitoren typische Nebenwirkungen, wie Hyperphosphatämie, auftraten [47, 85]. In einer Phase-I-Studie wurden 36 Patienten mit zuvor unbehandelten Mesotheliom mit der Ligandenfalle FP-1039 in Kombination mit Standard-Chemotherapie behandelt. Dabei konnte eine objektive Ansprechrate von 48 % erreicht werden [85]. In einer weiteren Phase-I-Studie mit 39 Patienten mit lokal fortgeschrittenen oder metastasierten Tumoren (darunter vier Patienten mit Kopf-Hals-Tumoren) konnte jedoch kein objektives Ansprechen beobachtet werden (NCT00687505). Eine primäre Selektion der Patienten anhand von Veränderungen des FGFR-Signalwegs erfolgte in dieser Studie nicht [47].

## Schlussfolgerung

Die molekulare Landschaft des HNSCC ist inzwischen umfangreich charakterisiert und zeigt hierbei charakteristische, therapeutisch adressierbare genomische Veränderungen. Zielgerichtete Therapien haben bisher nur eine geringe Wirksamkeit in unselektierten Kohorten von HNSCC-Patienten gezeigt. Aktuell basieren therapeutische Strategien noch auf Tumorlokalisation und Tumorstadium, während molekulare Differenzierung und Tumorbiologie noch keinen etablierten Stellenwert im Entscheidungspfad der individuellen Therapieplanung erlangen konnten. Eine Berücksichtigung der molekularen Eigenschaften individueller Tumoren bei der Wahl einer zielgerichteten Therapie, inklusive einer gegen den FGFR-Signalweg gerichteten Therapie, birgt das Potenzial, die Behandlung von HNSCC-Patienten zu verbessern [54].

Genomische Veränderungen innerhalb des FGFR-Signalwegs sind eine der häufigsten molekularen Eigenschaften von HNSCC. Insbesondere die Amplifikation der FGFR (v. a. *FGFR1*) und FGF (*FGF3, FGF4* und *FGF19*) sind häufig bei HPV-negativen HNSCC zu finden. Die hohe Prävalenz genomischer Veränderungen des Signalwegs und die biologische Relevanz lassen eine FGFR-gerichtete Therapie vielversprechend erscheinen. Erste Ergebnisse weniger FGFR-TKI-behandelter HNSCC-Patienten deuten auf eine Wirksamkeit hin. Wirksamkeit wurde darüber hinaus bei weiteren Tumorerkrankungen des Kopf-Hals-Bereichs (Schilddrüsenkarzinome, adenoid-zystische Karzinome) belegt. Eine gegen FGFR-gerichtete Therapie von Kopf-Hals-Tumorpatienten könnte daher zukünftig eine Rolle in dem Portfolio der Behandlungsoptionen spielen.

Bisherige Daten deuten darauf hin, dass die mRNA-Expression der FGFR einen prädiktiven Wert für das Ansprechen von HNSCC auf FGFR-TKI haben könnte. Allerdings ist die Datenlage hierzu noch nicht ausreichend, und weitere potenziell prädiktive molekulare Eigenschaften, wie beispielsweise FGFR-Mutationen, FGFR/FGF-Amplifikationen (insbesondere Chromosom 11q13 mit *FGF3, FGF4* und *FGF19*) und Kombinationen hieraus, sollten als potenzielle Stratifizierungsparameter in Erwägung gezogen und untersucht werden. Weitere gezielte Therapieansätze umfassen unter anderem die zielgerichtete Inhibition von FGFR4 bei Tumoren, die eine *FGF19*-Amplifikation/-Überexpression zeigen und/oder FGFR4 überexprimieren. Diese Therapien zeigen erste vielversprechende Ergebnisse im hepatozellulären Karzinom [86].

Seit 2017 prüfen umfassende biomarkergesteuerte Phase-II-Umbrella-Studien unter anderem den Einsatz von FGFR-TKI (nichtselektiv: Nintedanib, selektiv: Rogaratinib) im HNSCC (EORTC-Studie: NCT03088059 [54], TRIUMPH-Studie: NCT03292250). Die Ende 2020 bzw. Ende 2021 zu erwartenden Ergebnisse werden einen Einblick in das Potenzial von FGFR-gerichteten Therapien bei der Behandlung des lokal fortgeschrittenen oder rekurrenten/metastasierten HNSCC liefern.

## Fazit für die Praxis

- Eine Behandlung mit Erdafitinib oder Rogaratinib von Patienten mit rekurrentem oder metastasiertem HNSCC, für die es keine weiteren Therapieoptionen gibt, könnte wirksam sein, vor allem wenn genetische Alterationen der FGFR oder FGF vorliegen.
- Die FGFR-gerichtete Therapie von Patienten mit Kopf-Hals-Tumoren sollte im Rahmen eines molekularen Tumorboards diskutiert werden.
